# Accredited ocean sanctuaries for transforming captive cetacean care

**DOI:** 10.1371/journal.pbio.3003166

**Published:** 2025-05-20

**Authors:** Lori Marino, Charles Vinick, Katy Foster, Jeff Foster, Rob Hicks, Graham McGrath, John Racanelli, Janesse Brewer

**Affiliations:** 1 Whale Sanctuary Project, Washington, DC, United States of America; 2 Merlin Entertainments, London, United Kingdom; 3 SEA LIFE TRUST, London, United Kingdom; 4 National Aquarium, Baltimore, Maryland, United States of America; 5 23.4 Degrees, Dillon, Colorado, United States of America

## Abstract

Increasing concerns over the welfare of captive cetaceans (dolphins, whales, and porpoises) have led to calls for their transfer from marine parks and aquariums to sanctuaries. This Perspective describes an unprecedented collaboration to develop the first accreditation guidelines for authentic ocean sanctuaries to transform cetacean care.

## Teaser

Increasing concerns over the welfare of captive cetaceans (dolphins, whales, and porpoises) have led to calls for their transfer from marine parks and aquariums to sanctuaries. We describe an unprecedented collaboration to develop the first accreditation guidelines for authentic ocean sanctuaries to transform cetacean care.

More than 3,500 cetaceans live in captivity around the world [1], mostly in concrete tanks in entertainment parks. Captive cetaceans frequently experience compromised welfare in the form of stress-related physical and psychological illness, social abnormalities, and shorter lifespans [2–4]. They experience weakened immune system function resulting in opportunistic infections, as well as exhibit self-harming and other abnormal behaviors, e.g., stereotypies, and often have difficulty nurturing their calves within artificial social groupings in crowded tanks [2–4]. This welfare science, in combination with changing public opinion [5] as well as the closing of dolphin and whale exhibits worldwide, is driving the call for habitat and care alternatives for captive cetaceans who do not have other options due to regulatory requirements or a lack of survival skills to live in the wild. For example, The Dolphin Company’s recent filing for bankruptcy may necessitate the re-homing of 295 bottlenose dolphins from their 25 facilities across seven countries.

We propose that ocean-based sanctuaries for cetaceans, which more closely mirror the natural environment to which cetaceans have adapted over millions of years, could improve well-being. While the first generation of cetacean sanctuaries approach completion, we can draw on evidence from terrestrial sanctuaries and what we know about the health, well-being, and longevity of cetaceans in captive ocean environments and in the wild. Sanctuaries for other wild animals (elephants, great apes, big cats, etc.), in existence for decades, have provided evidence that improvement in health and well-being can be achieved in a more natural environment [6]. Dolphins in captive environments with more natural elements (e.g., sea pens or netted-off areas continuous with the ocean) display fewer behavioral abnormalities and may be less stressed than those living in tanks [7,8].

**Fig 1:**
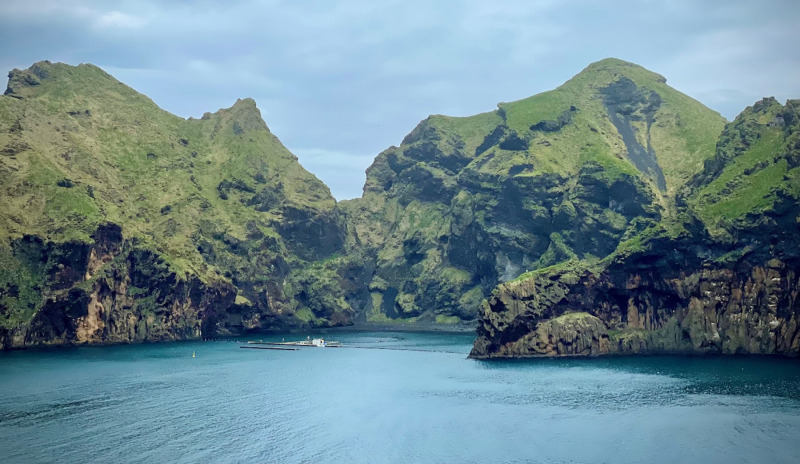
The SEA LIFE TRUST Beluga Whale Sanctuary. The sea sanctuary is located in Klettsvik Bay, Vestmannaeyjar, Iceland. Two lines of nets separate the part of the bay reserved for the TRUST’s beluga whales (approximately 32,000 m^2^), with an arrangement of pontoons in the center which provide docking access, animal care areas, and staff facilities. Connected to the pontoons is the “halo” intermediate habitat, a 50 m diameter/1,966 m^2^ area that staff can access around its entire circumference. Photograph by Graham McGrath.

A clear definition for what constitutes an authentic cetacean sanctuary, as well as standards that describe requirements, third-party monitoring and evaluation of those standards, are critically important for advancing the well-being of these large, highly intelligent, and socially complex mammals in sanctuaries. Here, we present and discuss the results of a unique collaboration across several organizations who are creating and completing cetacean sanctuaries (Whale Sanctuary Project, SEA LIFE TRUST, and the US National Aquarium). The Whale Sanctuary Project, founded in 2016, is a nonprofit organization comprising cetacean scientists, veterinarians, ex-trainers, managers, wildlife sanctuary experts, and advocates committed to transforming the way people relate to cetaceans by bringing an end to their exploitation and promoting seaside sanctuaries. The organization is currently creating an oceanside sanctuary for beluga whales and/or orcas in Nova Scotia. SEA LIFE TRUST is a global charity that advances conservation, wildlife sanctuaries, and education that inspires protection of the natural world. It has created a sanctuary for beluga whales in Klettsvik Bay, Iceland (see Fig 1) with an initial donation from Merlin Entertainment, and is preparing to introduce two beluga whales to the sanctuary in the near future. The US National Aquarium is a global thought leader in animal welfare, conservation, education, and community engagement. Its mission is to connect people with nature to inspire compassion and care for our ocean planet. In 2016 the National Aquarium announced its intentions to relocate its colony of bottlenose dolphins to a seawater sanctuary.

With the shared aim of creating a viable alternative to traditional captive cetacean entertainment facilities, we produced a foundational document for defining and implementing authentic cetacean sanctuaries and science-based professional accreditation guidelines which were adopted by the Global Federation of Animal Sanctuaries (GFAS) in 2023 (see the full guidelines here: GFAS-Cetacean-Standards-2023.pdf). The GFAS is the only global organization that provides accreditation for animal sanctuaries across a range of species and locations.

This collaboration defines signature characteristics of any authentic sanctuary as ‘place’, ‘principles’, and ‘practices’ that prioritize the well-being of each sanctuary resident over human interests. In addition to the care given in marine parks, i.e., food, protection, and veterinary services, authentic sanctuaries manifest the opportunity for superior welfare for the animals by allowing them to live in a setting more consistent with their evolutionary history and characteristics.

Fundamental to ‘place’ is a natural environment. Authentic sanctuaries, for all wild animals, are places designed to not only allow the residents to survive but, more importantly, to flourish [9]. Flourishing is a fundamental biological concept that underscores the importance of evolution and adaptation. In the case of cetaceans, sanctuary benefits include an ocean environment in the form of large coastal bays or equivalent ocean-based areas, more space, a more complex and dynamic natural environment, and the opportunity to interact with other species, e.g., fish, crabs, and other natural elements of their environment.

One of the greatest stressors in the lives of captive cetaceans is the inability to control fundamental aspects of their lives, e.g., where they go, how and what to eat, with whom they socialize, etc. [10]. Therefore, the foundational principle for all sanctuaries is to prioritize the well-being and autonomy of the residents. As there are no requirements for visitor entertainment, interaction, or other commercial aspects of traditional marine parks, cetacean sanctuaries are in the unique position of providing an opportunity for the residents to spend their days largely unfettered by human interests (apart from human care).

And, finally, practices in authentic sanctuaries are consistent with traditional marine parks in terms of providing high-quality veterinary care, regular health assessments, food, and safety. However, an important distinction is that in authentic sanctuaries breeding is prevented—not because reproduction and raising young aren’t important natural behaviors, but because sanctuaries are committed to not perpetuating wild animal captivity.

Authentic cetacean sanctuaries represent a model for a new relationship between the public and cetaceans, fostering learning and respect for wild animals and the natural settings they evolved to live and flourish in. For example, sanctuaries can provide unique opportunities for veterinary teaching and non-invasive/non-intrusive research on cetacean health and behavior in a natural setting more applicable to research practices in the wild. The cetacean sanctuary model is still a work in progress, and much will be learned and likely adjusted as the first of these sanctuaries open. It is anticipated that as we learn more about the complex needs of cetaceans in sanctuaries, the GFAS accreditation standards will be re-considered periodically in the same way that accreditation standards for terrestrial sanctuaries, zoos, and marine parks have over time. However, the creation of these standards advances accountability and transparency about what constitutes an authentic sanctuary. And sanctuaries hold the promise of achieving a shared goal—better well-being for captive wild animals and a more authentic understanding and respectful relationship to these intelligent, complex mammals.

## Abbreviation

**:**
GFAS: Global Federation of Animal Sanctuaries
